# Supplementary material for: A Global Screen for Assembly State Changes of the Mitotic Proteome by SEC-SWATH-MS
Source: Cell Syst. 2020 Feb 26;10(2):133–155.e6. doi: 10.1016/j.cels.2020.01.001 (PMC7042714; doi:10.1016/j.cels.2020.01.001)

Q8TAG9 | EXOC6_HUMAN | EXOC6 SEC15A SEC15L SEC15L1
Monomer MW [kDa]: 93.722 Monomer expected elution fraction: 41

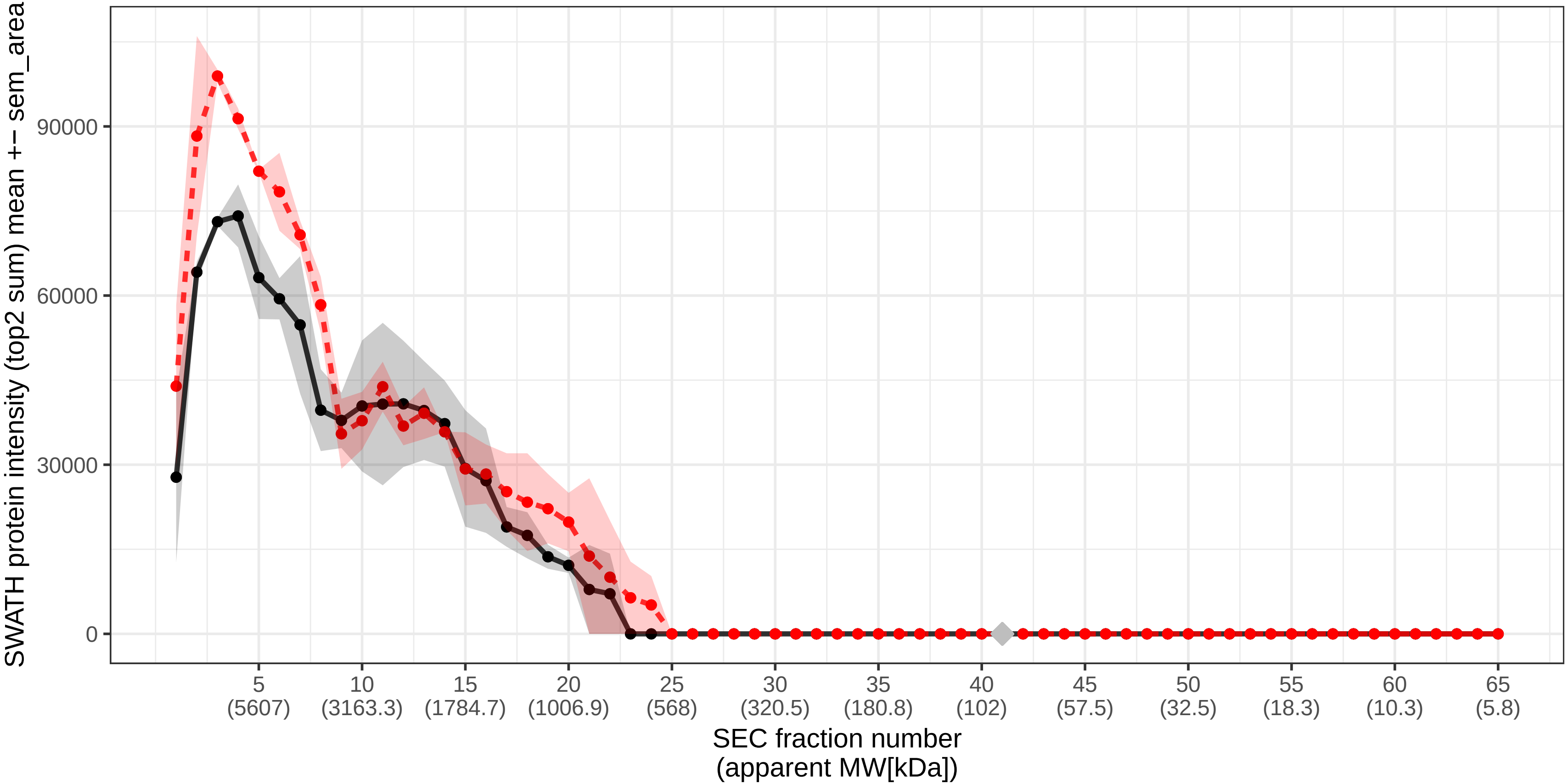

Supplement: Data S1. SEC-SWATH-MS Protein Chromatograms, Related to Figure 1 [file mmc6.zip › SECchrom_Q8TAG9_EXOC6_HUMAN_EXOC6_SEC15A_SEC15L_SEC15L1.pdf]
